# The Efficacy of Psychedelic-Assisted Therapy in Managing Post-traumatic Stress Disorder (PTSD): A New Frontier?

**DOI:** 10.7759/cureus.30919

**Published:** 2022-10-31

**Authors:** Arafath Mohamed, Shehla Touheed, Muzammil Ahmed, Mosab Hor, Sara Fatima

**Affiliations:** 1 Critical Care Medicine, Asian Institute of Gastroenterology (AIG) Hospitals, Hyderabad, IND; 2 Medicine, Shadan Institute of Medical Sciences, Hyderabad, IND; 3 Critical Care Medicine, Olive Hospital, Hyderabad, IND; 4 Ophthalmology, Children Retina Institute, Los Angeles, USA; 5 Emergency Medicine, Asian Institute of Gastroenterology (AIG) Hospitals, Hyderabad, IND

**Keywords:** post traumatic stress disorder, ptsd, hallucinogens, dmt, cannabinoids, psilocybin, ketamine, lsd, psychedelic-assisted therapy, mdma

## Abstract

Post-traumatic stress disorder (PTSD) is a significant public health concern for which existing therapies are only marginally effective. Indisputably, the primary line of treatment for PTSD is psychotherapy, according to current treatment guidelines. However, PTSD continues to be a chronic condition even after psychotherapy, with high psychiatric and medical illness rates. There is a dire need to search for new compounds and approaches for managing PTSD. The usage of psychedelic substances is a potential new method. This article reviews the efficacy of psychedelic-assisted therapy in treating PTSD and improving patient outcomes. It will examine current research on the topic and evaluate the benefits and drawbacks of different therapies. The current evidence for the use of four different types of psychedelics (3,4-methylenedioxymethamphetamine, ketamine, classical psychedelics, and cannabis) in the treatment of PTSD will be reviewed. It will also include an overview of the therapeutic justification, context of use, and level of evidence available for each drug. Several questions are formulated that could be studied in future research in order to gain a better understanding of the topic.

## Introduction and background

Post-traumatic stress disorder (PTSD) is a complex mental disorder that affects 7.7 million adults in the United States annually, with one in 11 individuals being diagnosed with PTSD at some point in their lives. More than twice as many women (10%) as men (4%) have PTSD, with sexual assault being the most common traumatic incidence [1]. According to the DSM-5 criteria, PTSD is a mental health disorder that can develop after exposure to a traumatic event, such as death, serious injury, or sexual violence [2]. PTSD is characterized by recurring and distressing symptoms that last at least one month after the traumatic event. Symptoms of PTSD include dissociative reactions, distressing dreams, avoidance of trauma-related stimuli, perpetual psychological distress, adverse changes in cognition and mood, and variation in arousal and reactivity [3]. 

The selective serotonin reuptake inhibitors (SSRIs) sertraline and paroxetine are U.S. Food and Drug Administration (FDA)-approved first-line therapeutics for the treatment of PTSD [2]. It is estimated that 40-60% of patients treated with these compounds do not experience any response [4]. Although trauma-focused psychotherapies, such as prolonged exposure and cognitive behavioral therapy, are considered the most effective treatments for PTSD, many people do not respond well to these treatments or continue to have significant symptoms, and dropout rates are high [4]. Poor outcomes from treatment are often associated with comorbid conditions such as childhood trauma, alcohol and substance abuse, depression, and dissociation [2]. Therefore, it is essential to identify a beneficial treatment for those typically resistant to treatment.

Psychedelic-assisted therapy involves a different approach to treating PTSD. It includes an evaluation by a medical professional to ensure safety, a few sessions to prepare the patient and build rapport, and one to three (six to eight hours) sessions where the patient is under the psychedelic influence [5]. Finally, there are integration sessions where the experience is processed and explored [6]. The positive outcomes of these intensive treatments are likely due to the intensity of the interventions themselves [5]. They also require a lot of resources, so careful consideration must be given to how they can be integrated into the existing healthcare systems in a way that is fair and accessible to everyone [6].

The exact mechanisms by which psychedelics produce changes in perception and cognition are not fully understood. However, they may involve reduced activity in the brain's default mode network and increased functional connectivity between different brain regions [5]. The neural networks (cingulate, insular, prefrontal cortex, amygdala, and hippocampus) that involve the monoamine neurotransmitters like serotonin, dopamine, and glutamate are altered in PTSD due to trauma and potential genetic susceptibility. The endocrine and autonomic nervous systems are subsequently impacted by these network alterations, which results in the physiological and subjective symptoms of PTSD. The neural network and subjective levels of this process are where psychedelic-assisted therapy has the most observable effects [7].

**Figure 1:**
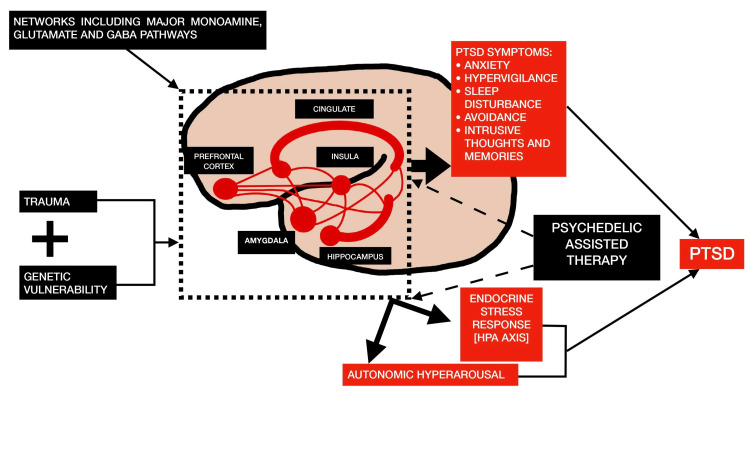
The hypothesized impact of psychedelic-assisted therapy on the pathophysiology of PTSD PTSD - post-traumatic stress disorder; HPA - hypothalamic-pituitary-adrenal

Figure 1 illustrates the hypothesized impact of psychedelic-assisted therapy on the pathophysiology of PTSD [7].

There has been a recent resurgence of interest in psychedelics as potential medicines representing a new paradigm in drug development. Although there are still many unanswered questions about how these drugs work and how effective they are, there has been a reawakening of interest in their potential therapeutic benefits for conditions ranging from depression and substance abuse to PTSD [4].

This traditional review article will discuss the efficacy of psychedelic-assisted therapies in the treatment of PTSD and in improving patient outcomes. It will explore the current research on the subject and assess the pros and cons of these therapies. For this review, we use a broad definition of psychedelic drugs, including 3,4-methylenedioxymethamphetamine (MDMA), ketamine, and cannabis, whose pharmacological profiles differ significantly from the serotonergic classical psychedelics (such as psilocybin and lysergic acid diethylamide). However, they all have the ability to induce altered states of consciousness. 

In the sections that follow, we will give a general overview of the evidence that is currently available for four different types of psychedelics (MDMA, ketamine, classical psychedelics, and cannabis), as well as background information on the therapeutic justification for each drug, the context in which it is used, and the level of evidence that is currently available for the treatment of PTSD.

Search strategy 

We comprehensively searched PubMed, Google Scholar, and ScienceDirect databases for English-language literature. Extensive research was conducted using keywords to validate the studies analyzing and assessing the efficacy of psychedelic-assisted therapies in managing PTSD. Keywords included lysergic acid diethylamide (LSD), MDMA, ketamine, psilocybin, PTSD, psychedelic-assisted therapy, hallucinogens, cannabinoids, dimethyltryptamine (DMT), and synaptic connectivity. All the articles were considered without the restriction of time of publication or study type, i.e., traditional reviews, systematic reviews, clinical trials, case-control studies, and cohort studies. Studies were not refined based on age and ethnicity, and the search had no demographic limitations. Animal studies were excluded. As this is a traditional review article, Preferred Reporting Items for Systematic Reviews and Meta-Analyses (PRISMA) guidelines were not followed. Data was collected from inception up to October 2022.

## Review

MDMA

MDMA (3,4-Methylenedioxymethamphetamine) was first created in 1912 as a part of the process of making medicine that helps control bleeding. It was not widely known until the 1970s when its psychoactive effects were discovered. It gained the attention of many psychotherapists, who started using it as a tool for psychotherapy. It became widely known as a "party drug" in 1985, after which the U.S. Drug Enforcement Administration placed it on schedule 1 of the Controlled Substances Act, making its therapeutic use illegal. Despite its initial therapeutic use, no clinical trials were conducted until 2000 [8]. After this, it has been studied as a potential treatment for PTSD [9], alcohol use disorder [10], and social anxiety in autistic adults [11].

Several scientific papers have discussed the potential therapeutic benefits of using MDMA as part of psychotherapy [12]. It has been demonstrated that MDMA increases prosocial behavior, affective contact pleasure, and emotional empathy [13]. Additionally, it has been demonstrated to raise subjective judgments of openness, trust, and interpersonal closeness [14]. From a neurobiological perspective, MDMA has been shown to reduce activity in the amygdala, a region of the brain associated with fear and anxiety, while increasing activity in the frontal cortex, a region involved in higher-level thinking and decision-making. This may benefit people with PTSD, as they often have impaired frontal cortex activity [15].

Six women with chronic PTSD brought on by sexual assault participated in the first clinical trial examining MDMA-assisted psychotherapy for treating PTSD, which took place in Spain from 2000 to 2002 [16]. Patients underwent several 90-minute non-drug psychotherapy sessions before and after low doses of MDMA (50 or 75 mg). PTSD symptom reductions were seen in both MDMA groups. However, statistical analysis was not possible due to the limited patient population. The most significant finding from this study was that giving MDMA to this demographic appeared to be both physically and mentally safe [16].

The first randomized placebo-controlled trial (RCT) of MDMA-assisted psychotherapy for PTSD was published in 2010 [9]. In a study of treatment-resistant patients, those who received two sessions of MDMA (plus an optional booster of 62.5 mg) had better outcomes than those who received a placebo. According to the study results, 83% of the patients given MDMA no longer met the criteria for PTSD, as opposed to only 25% of the patients in the placebo group [9]. The long-term follow-up results revealed that treatment effects persisted for three years [17]. These findings were also confirmed in other studies where individuals receiving MDMA also displayed increases in post-traumatic growth [18]. A pooled analysis of data from six different RCTs involving 105 patients revealed that those who received MDMA experienced significantly greater reductions in symptoms of PTSD than those in the control group. After two MDMA sessions, 54.2% of patients no longer met the diagnosis of PTSD, compared with 22.6% in the control group [12]. According to studies comparing the efficacy of paroxetine and sertraline with that of MDMA-assisted psychotherapy, the latter has greater effect sizes and lower dropout rates. Based on these findings, the U.S. Food and Drug Administration granted MDMA the status of breakthrough therapy for the treatment of PTSD [19].

The side effects of MDMA that users most commonly reported include anxiety, a tight feeling in the jaw, headache, and fatigue. When the first effects of MDMA become apparent, anxiety episodes can occur but are readily managed with psychotherapy treatment [12]. MDMA's effects on heart rate and blood pressure increase in severity as the dosage increases. Some forms of hypertension and severe cardiovascular pathology are considered to be conditions that would make this medication inadvisable [20]. As induced by MDMA, slight hyperthermia presents no problem when used in a medical setting [21]. Concerns about MDMA's potential for abuse and neurotoxicity have been voiced by certain authors [22]. However, the usage of MDMA under medical supervision has prevented neither of these from happening [12].

Table 1 provides a review of the clinical trials undertaken on the use of MDMA in the management of PTSD.

**Table 1:** Summary of clinical trials conducted on the use of MDMA in PTSD MDMA - 3,4-Methylenedioxymethamphetamine; PTSD - post-traumatic stress disorder

Author	Study type	Study population	Conclusion
Mithoefer et al. (2018) [18]	Randomized, double-blinded control trial	Twenty-six first responders/veterans with chronic PTSD. Seven received 75 mg MDMA-assisted psychotherapy. Twelve received 125 mg MDMA-assisted psychotherapy. Seven received placebo-assisted psychotherapy.	The findings of this study suggest that MDMA doses as low as 75 mg may help ease the symptoms of PTSD. Additionally, it expands on MDMA's therapeutic success in treating chronic PTSD and shows promising outcomes in a particular patient group.
Mithoefer et al. (2013) [17]	A long-term follow-up study to randomized, double-blinded control trial	Sixteen participants completed the second assessment. Evaluated both the experimental group and the placebo group after they switched to get MDMA therapy.	The follow-up study shows that MDMA treatment may have long-term effects and be sustainable. The study also questions whether it is necessary to have a third treatment session to achieve a clinical response.
Mithoefer et al. (2011) [9]	Randomized, double-blinded control trial	Twenty participants with treatment-resistant PTSD. Twelve received MDMA-assisted psychotherapy. Eight received placebo-assisted psychotherapy only.	The study showed that MDMA-assisted psychotherapy could be an effective treatment for PTSD.
Bouso et al. (2008) [16]	Randomized, double-blinded control trial	Six women with treatment-resistant PTSD. Four with MDMA-assisted psychotherapy. Two with psychotherapy only.	There is not enough data from the study to make any firm conclusions. More research is needed to see if MDMA can assist with treatment-resistant PTSD.

Ketamine

Ketamine, a noncompetitive N-methyl-D-aspartate-receptor antagonist, was first synthesized in 1962. It was approved as an anesthetic in 1970 and is often referred to as a "dissociative psychedelic" [23]. Based on an aversion strategy, ketamine-assisted psychotherapy has been utilized to treat alcoholism and heroin addiction since the 1990s [24]. Research on ketamine has grown in the last 20 years as a treatment option for various psychiatric conditions [25]. Multiple clinical studies at the beginning of the early 2000s revealed ketamine's rapid antidepressant effects [26]. Studies examining its antidepressant benefits [26] and its implications on suicidal ideation [27] have increased exponentially since then. It is also a candidate for focusing on emotional memories and is being researched more extensively for the treatment of PTSD [28].

Several neurobiological processes have been proposed for how ketamine might lessen the symptoms of PTSD [29]. One theory suggests that PTSD is a "synaptic disconnection syndrome". Psychedelic drugs like ketamine may work by rapidly increasing the brain's ability to form new connections between neurons. This could explain why these drugs have therapeutic effects [30].

In 41 patients with chronic PTSD and related depressive symptoms, the only RCT on ketamine for the treatment of PTSD compared a single IV infusion of 0.5 mg/kg ketamine with a single IV infusion of 0.045 mg/kg midazolam [31]. The severity of PTSD symptoms was significantly and quickly reduced by ketamine infusion, and this effect persisted for seven days following the single infusion. These transient improvements suggest a rapid neurobiological operating mechanism that is also seen when ketamine is used to treat depression [31]. Research indicates that ketamine's therapeutic effects on PTSD can be strengthened and prolonged with repeated infusions, similar to how it is used to treat depression [32]. In another study, 15 war veterans with comorbid PTSD and treatment-resistant depression received six intravenous ketamine infusions (0.5 mg/kg) throughout 12 days. The median time to relapse was 41 days, and the remission rate for PTSD was 80% [32].

So far, only one published study has tried using ketamine to treat PTSD using a similar strategy to drug-assisted psychotherapy [33]. This study combined the administration of ketamine with mindfulness-based cognitive therapy in order to treat patients with refractory PTSD. Patients received a single intravenous injection of 0.5 mg/kg ketamine or saline over 40 minutes. Before the treatment, patients were asked to think about their traumatic experience in a controlled way by reflecting on a personalized story about what happened. In addition to the infusion, they practiced mindfulness exercises for two cycles (ten minutes each). Ketamine enhanced a state in which patients passively welcomed the unpleasant memories as they came rather than reacting fearfully to them. Patients who received ketamine had a much longer-lasting improvement (34 days) in PTSD symptoms than those who received a placebo (16 days) [33].

Drowsiness, nausea, dizziness, altered vision, altered perception, and dose-dependent dissociative symptoms are among the most reported ketamine adverse effects [34]. In some cases, ketamine can cause transient anxiety [35]. Clinical settings that are supportive can minimize such reactions [35]. Ketamine has sympathomimetic effects, which raise blood pressure and heart rate. As a result, severe cardiovascular disease and various types of hypertension are considered contraindications [34]. While the early psychedelic effects of ketamine are frequently seen as adverse side effects, some of these effects are thought to have therapeutic benefits from a substance-assisted psychotherapy approach [33].

Classical psychedelics

Psilocybin, lysergic acid diethylamide (LSD), and N, N-dimethyltryptamine (DMT) are a few of the substances that make up the class of drugs known as "classical psychedelics", and they all work primarily by agonistic activity at the 5-HT2A receptor [4]. Psilocybin has been studied to treat depression, anxiety [36], tobacco [37], and alcohol addiction [38]. Results of several recent studies point to the possibility that PTSD treatment may benefit from the effects of classical psychedelics [4]. It has also been demonstrated that classical psychedelics reduce the amygdala's responsiveness when processing emotions [39]. This can help PTSD patients as they frequently exhibit increased amygdala activation [40]. They may also play a therapeutic role in the management of PTSD by virtue of other acute effects, such as higher levels of emotional empathy [41], increased insightfulness [42], improvements in acceptance [43], and emotive transformational events, which has shown to be a key mediator in the long-term psychological change in other mental disorders [44]. So far, no clinical trials have been conducted to examine the potential of classical psychedelics for the treatment of PTSD [4].

Classical psychedelics can occasionally cause brief episodes of nausea, vomiting, and discomfort in the body [36]. They also cause mentally challenging experiences, such as anxiety and confusion [45]. Some patients may exhibit emotional vulnerability in the days after the therapy, which emphasizes the significance of receiving psychological assistance [43]. Classical psychedelics can cause an increase in heart rate and blood pressure. Therefore, some forms of hypertension and severe cardiovascular pathology are contraindications. When used under medical supervision, they are not harmful to the human body and do not result in dependence or significant side effects (such as flashbacks) [45].

Cannabinoids 

For thousands of years, people in Asia and the Middle East have used cannabis for religious, therapeutic, and other purposes [46]. It was first used medically in the West in the 19th century to treat rheumatism, convulsions, and other conditions [47]. Since the discovery of the endogenous cannabinoid system in the 1990s, scientific research into the medical uses of cannabinoids has grown [46]. The endocannabinoid system is being extensively researched as a potential therapeutic target for the treatment of PTSD [48]. In the past 20 years, several nations have legalized medical cannabis. Recently, the World Health Organisation (WHO) proposed that cannabis be rescheduled to allow for medical applications [49].

There are more than 100 distinct cannabinoids found in cannabis. However, tetrahydrocannabinol (THC) and cannabidiol (CBD) are the two most researched [50]. In addition, research has also been done on several synthetic cannabinoids, such as nabilone and dronabinol [51]. Cannabinoids interact with the endocannabinoid system, which is responsible for emotional memories and mediating the hypothalamic-pituitary-adrenal response to stress [52]. Chronic stress has been shown to decrease the number of cannabinoid type 1 receptors [53], which may contribute to symptoms of PTSD such as hyperarousal, sleeping problems, and intrusive memories [46]. These results lend credence to the use of cannabis in the management of PTSD.

In contrast to other psychedelic drugs, cannabis and synthetic cannabinoids are primarily utilized and researched to temporarily relieve PTSD symptoms. They may also have value in substance-assisted psychotherapy [4]. THC and CBD are two cannabinoids that have demonstrated the ability to promote fear extinction [54] and to hinder the reconsolidation of fear memories [55]. The efficacy of exposure therapy might be improved by the tailored use of certain cannabinoids since fear extinction processes are crucial for successful exposure therapy [48] and because PTSD patients have demonstrated poorer fear extinction learning and recall than controls [56].

The use of cannabis to treat PTSD has been examined in several research studies. In one study, ten outpatients already receiving stable medication for their PTSD symptoms were given 5 mg of THC (sublingually) twice daily as an adjunctive treatment [57]. Overall symptom severity, sleep quality, nightmare frequency, and hyperarousal symptoms all significantly improved. Because of the limited sample size, lack of a control group, and interference from other drugs, these results should be viewed with caution [57].

In a cross-over, placebo-controlled trial, nabilone was administered to ten people living with PTSD from the Canadian military who had dreams connected to traumatic events [58]. Patients who took nabilone for seven weeks demonstrated considerably more substantial improvements in their PTSD symptoms based on their Clinician-Administered PTSD Scale (CAPS) Recurring and Distressing Dream scores, Clinical Global Impression of Change scores, and the General Well-Being Questionnaire scores compared to when they got a placebo [58]. These results support the preliminary findings from previous nabilone studies in 47 PTSD patients who participated in an open-label chart review study [59] and 104 male inmates who participated in a retrospective study [60], both of which found that the drug had positive effects on a number of PTSD symptoms, particularly nightmares and sleep issues. High-quality studies are needed to further investigate the effects of cannabis on PTSD, according to a new meta-analysis [61].

Cannabinoids frequently cause adverse effects, including lethargy, disorientation, and dry mouth [62]. Vomiting and nausea may occasionally happen during heavy use. Cannabinoids can cause anxiety, mainly if used in more significant amounts [63]. They may also increase the chance of developing psychotic illnesses in those predisposed to them [64]. Regular cannabis usage for recreational purposes has also been linked to cognitive deficiencies, particularly in adolescents [63]. The emergence of cannabis use disorder in susceptible individuals is another possible risk factor [65]. This highlights the significance of appropriate screening and treatment monitoring.

Limitations

Classical psychedelics such as psilocybin and LSD have demonstrated encouraging outcomes for treating several psychiatric conditions. However, the limitations of this study have shown that PTSD-specific clinical trials are still scarce. There is a need for more extensive randomized controlled trials to assess the safety and efficacy of cannabinoids, particularly whole-plant cannabis. There are many unanswered questions regarding appropriate doses, ratios of cannabinoids, methods of administration, long-term risks, and side effects. In most recent research, therapists observed each patient's internal process as it developed throughout their medication sessions in a rather non-directive manner. It is still unclear if individuals who are experiencing the effects of psychedelics might potentially benefit from more direct methods like extended exposure, cognitive processing therapy, or eye movement desensitization and reprocessing.

## Conclusions

There is no doubt that exposure-based psychotherapy should be used as the first line of treatment for PTSD. However, PTSD frequently persists as a chronic condition with high rates of psychological and medical comorbidity. Novel therapies that might improve the effectiveness of PTSD treatments are thus urgently needed. As this review highlights, psychedelic substances provide prospects for a revolutionary method of treating PTSD. Each of the examined substances has a distinctive potential, from their use to quickly target PTSD symptoms to their use as adjuncts to support psychotherapy. More research is needed to determine the safety and efficacy of psychedelics and identify the patients for whom these treatments might be most effective. Another crucial topic for investigation is contraindications concerning particular symptom clusters and personality traits. From a therapeutic and neurobiological standpoint, there is also a need to better comprehend the psychological states these psychedelic substances can induce. These new research studies will enable us to assess how these alterations may improve the psychotherapeutic treatment of PTSD and better comprehend the physiological mechanisms of action.
